# Molecular Detection of Human Papillomavirus (HPV) and Other Sexually Transmitted Pathogens in Cervical and Self-Collected Specimens

**DOI:** 10.3390/ijms26031296

**Published:** 2025-02-03

**Authors:** Chiara Giubbi, Marianna Martinelli, Michelle Rizza, Maria Letizia Di Meo, Ruth Chinyere Njoku, Federica Perdoni, Giulio Mannarà, Rosario Musumeci, Robert Fruscio, Fabio Landoni, Clementina Elvezia Cocuzza

**Affiliations:** 1 School of Medicine and Surgery, University of Milano-Bicocca, 20100 Milan, Italy; chiara.giubbi@unimib.it (C.G.); m.rizza8@campus.unimib.it (M.R.); r.njoku@phd.uniss.it (R.C.N.); federica.perdoni@unimib.it (F.P.); giulio.mannara@unimib.it (G.M.); rosario.musumeci@unimib.it (R.M.); robert.fruscio@unimib.it (R.F.); fabio.landoni@unimib.it (F.L.); clementina.cocuzza@unimib.it (C.E.C.); 2 Fondazione IRCSS San Gerardo dei Tintori, 20900 Monza, Italy; marialetizia.dimeo@gmail.com; 3 Department of Biomedical Science, University of Sassari, 07100 Sassari, Italy

**Keywords:** high-risk human papillomavirus (hrHPV), sexually transmitted infections (STIs), self-sampling

## Abstract

This study investigated the detection of high-risk Human Papillomavirus (hrHPV) and seven other pathogens associated with sexually transmitted infections (STIs) in matched clinician-collected cervical samples and self-taken vaginal and urine specimens collected from 342 asymptomatic women referred to colposcopy to evaluate (i) the concordance in the molecular detection of investigated pathogen in three different sample types; (ii) the analytical sensitivity and specificity of STIs detection on self-samples; and (iii) the distribution of STIs in hrHPV-positive and hrHPV-negative women. Pathogens detection was performed using Anyplex™II HR and Anyplex™II STI-7e, respectively. Good/substantial agreement was observed between cervical and self-taken samples in detecting hrHPV (κ = 0.870 and κ = 0.773 for vaginal and urine). The agreement between cervical and self-taken samples for detecting STIs was found to be significant (κ = 0.779 and κ = 0.738 for vaginal and urine), with almost perfect agreement between urine and vaginal specimens (κ = 0.899). The positivity rate for all investigated STIs was found to be higher in hrHPV-positive compared to hrHPV-negative women. In conclusion, self-sampling proved to be a valid alternative to cervical samples to detect hrHPV and STIs, but further studies are required to evaluate the role of STI coinfections in cervical lesions development and progression.

## 1. Introduction

Cervical cancer represents a serious threat to women’s health globally, with an age-standardized incidence rate of 14.1 per 100,000 women. It is the fourth most common cancer among women, with 661,000 new cases and 348,000 deaths reported in 2022, according to GLOBOCAN data [1]. Persistent infection with high-risk Human Papillomavirus (hrHPV) is widely acknowledged as the primary cause of cervical cancer and associated with a large percentage of anal cancers and cancers of the vulva, vagina, penis and oropharynx [2].

To date, nearly 200 different HPV types have been identified, including 12 classified by the International Agency for Research on Cancer (IARC) as oncogenic or “high-risk” HPV types belonging to the Group 1 (HPV16, 18, 31, 33, 35, 39, 45, 51, 52, 56, 58 and 59), with HPV16 and HPV18 being the two genotypes more frequently associated with cervical cancer development [3].

Presently, molecular testing for the detection of HPV nucleic acids is gradually replacing cytology in cervical cancer screening and more than 200 HPV tests are commercially available, using different technologies and targeting different HPV genotypes. Some of them are full genotyping assays detecting each HPV type individually; others are partial, extended or without genotyping [4]. Only few tests are validated for the use in cervical cancer prevention according to internationally recognized criteria [4].

The use of self-samples is currently being promoted in cervical cancer prevention worldwide to improve the participation of women in screening programs [5,6]. Self-collection, a non-invasive and easy-to-perform procedure, may attenuate cultural and/or socio-economic barriers, increasing the participation in prevention programs of underserved cohorts [7,8,9].

The use of PCR-based HPV assays on self-collected samples has been described as having similar accuracy to that of clinician-collected cervical specimens [10,11], as also reported in a previous study by our group [12]. Recently, the VALHUDES (Validation of Human Papillomavirus Assays and Collection Devices for Self-samples and Urine Samples) protocol has been developed to evaluate the clinical accuracy of HPV tests in combination with self-collection devices [13]. Self-collection may represent a convenient way to screen women for other sexually transmitted infections (STIs), increasing the uptake of testing services compared to samples collected by healthcare professionals [14].

The increased prevalence of STIs reported by the European Centre for Disease Prevention and Control (ECDC) [15], urges the scientific community to consider new strategies to implement STI screening, diagnosis and treatment. In particular, in women, STIs are frequently asymptomatic, which, if untreated, can result in the persistence and/or spread of infections through sexual contacts [16]. The delayed diagnosis and treatment of STIs can also increase the risk of long-term health complications, including pelvic inflammatory disease and infertility in women [14]. The diagnosis of STIs usually requires screening and/or diagnostic procedures, which may be difficult to implement due to the social stigma associated with these infections and the limited access to healthcare, particularly in low–middle-income countries [9,16].

Interactions between HPV and other microorganisms sharing similar anatomical sites may be associated with the persistence of HPV infection and an increased risk of disease progression. However, the role of coinfections as a risk factor for the development of cervical cancer requires further investigation [17,18,19]. Some studies reported that *Chlamydia trachomatis* (*CT*) persistent infection may be associated with DNA damage due to reactive oxygen species increasing the risk of carcinogenesis related to hrHPV [18,20,21]; others indicated that *CT* may increase the risk of acquiring hrHPV infection by the disruption of cadherin–catenin junctions in cervical epithelial cells [22]. Smith and colleagues reported an association between *CT* infection and squamous cells intraepithelial cervical cancer [23], while Castle and colleagues did not find any association with the severity of cervical neoplasia [24]. The chronic inflammation caused by *Ureaplasma* spp. and *Mycoplasma* spp. seems to permit the entry of other pathogens or induce genetic alterations that might lead to the carcinogenesis of epithelial cells [25,26,27].

The present study aimed at evaluating the accuracy in terms of analytical sensitivity, specificity and agreement of self-collected vaginal and urine specimens, compared to clinician-collected cervical samples, for the detection of 14 hrHPV genotypes (HPV16, 18, 31, 33, 35, 39, 45, 51, 52, 56, 58, 59, 66 and 68) and seven other sexually transmitted microorganisms (*Ureaplasma parvum* (*UP*), *Ureaplasma urealyticum* (*UU*), *Mycoplasma genitalium* (*MG*), *Mycoplasma hominis* (*MH*), *Chlamydia trachomatis* (*CT*), *Neisseria gonorrhoeae* (*NG*), and *Trichomonas vaginalis* (*TV*)). In addition, we aimed to investigate the distribution of STIs among hrHPV-positive and hrHPV-negative women to evaluate the possible association of hrHPV-STI coinfections with the severity of cervical dysplasia.

## 2. Results

### 2.1. Study Population

Three hundred and forty-five women with a prior abnormal Pap smear were recruited at the first colposcopy referral visit. Their median (interquartile range, IQR) age was 37 (29–46) years. In total, 25.2% of women were younger than 30 years, 31.3% were aged 30–40, 28.7% were aged 41–50, 12.5% were aged 51–60 and 2.3% were older than 60 years. The cytology results show that 47.5% of women had low-grade squamous intraepithelial lesion (LSIL), followed by those with atypical squamous cells of undetermined significance (ASCUS) (24.9%) and those with high-grade squamous intraepithelial lesion (HSIL) (13.6%). The colposcopy examination showed positive findings in 127 patients, while 218 patients had negative findings. Patients underwent cervical biopsy and/or treatment with conization according to clinical judgment and local clinical protocols. The histological results showed four cases of cervical cancer: two cases of squamous cell carcinomas, one of adenocarcinoma “in situ” and one cervical carcinoma with dual histological components (Table 1).

### 2.2. HPV Detection and Genotyping

Among the enrolled study population (*n* = 345), three women were excluded from the analysis because one of their matched samples tested invalid twice. As a result, 342 women were included in the study.

**Figure 1:**
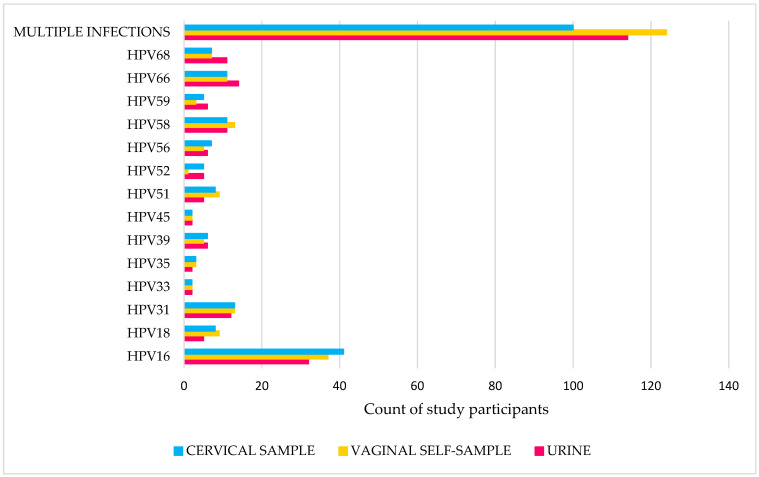
Prevalence of hrHPV genotypes in different samples.

The prevalence of hrHPV, in this cohort of women referred to colposcopy for abnormal cervical cytology, was 67.0% (229/342), 71.3% (244/342), and 68.1% (233/342) in cervical, vaginal, and urine samples, respectively, with HPV16 and HPV31 being the most common types, followed by HPV58 and HPV66 (Figure 1).

In total, 56.3% (129/229), 49.2% (120/244) and 51.1% (119/233) of the women showed a single infection with only one hrHPV type in the cervical swab, self-collected vaginal and urine samples, respectively, whereas multiple hrHPV infections were detected in 43.7% (100/229), 50.8% (124/244) and 48.9% (114/233) of patients in the cervical samples, vaginal samples and urine samples, respectively (Supplementary Table S1). No statistical difference was found in single and multiple infections rates between sample types (Supplementary Table S2).

### 2.3. Analytical Sensitivity, Specificity and Type-Specific Agreement of Self-Collected Samples for hrHPV Detection

**Figure 2:**
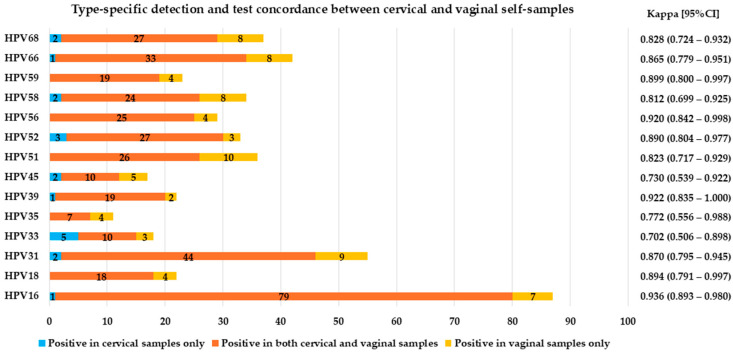
Type-specific detection and test concordance between cervical and vaginal self-samples.

**Figure 3:**
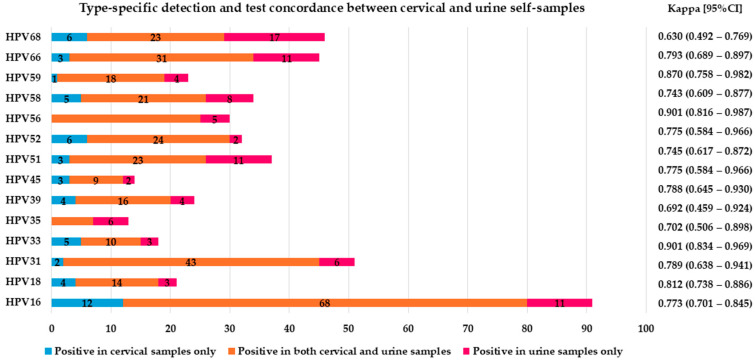
Type-specific detection and test concordance between cervical and urine self-samples.

The analytical sensitivity and specificity for the detection of any hrHPV in vaginal self-collected specimens relative to the results on cervical samples were 99.1% and 85.0%, respectively, while those in urine were 93.4% and 83.5%. Good/substantial agreement was observed between cervical and self-taken samples in detecting hrHPV (agreement 94.4% with κ = 0.870 and agreement 90.1% with κ = 0.773 for vaginal and urine specimens, respectively) (Figure 2 and Supplementary Table S3; Figure 3 and Supplementary Table S4).

### 2.4. STI Prevalence

In total, 47.9% (164/342), 57.9% (198/342), and 56.4% (193/342) of investigated women resulted positive for at least one of the seven STI-related pathogens under investigation in their cervical, vaginal and urine samples, respectively.

*Ureaplasma parvum* was the most prevalent pathogen in all samples, followed by *UU* and *MH*. No women showed infection with *NG*. In general, for most of the sexually transmitted pathogens, the detection rate was lower in cervical swabs (Table 2 and Supplementary Figure S1). Still, statistically significant differences using the X^2^ test with Yates correction were only found for *UP* positivity rates in vaginal (*p* < 0.01) and urine (*p* < 0.05) specimens compared to cervical samples (Supplementary Tables S5 and S6).

The majority of women with positivity for the STIs molecular panel were infected by a single microorganism in all sample types (cervical samples: 74.4%; vaginal self-collected samples: 72.2%; and urine: 73.1%) (Supplementary Tables S7 and S8).

### 2.5. Analytical Sensitivity, Specificity and Agreement of Self-Collected Samples for STIs Detection

Considering the results of the clinician-collected cervical samples as reference, the analytical sensitivity and specificity for the detection of any of the STIs of vaginal self-collected specimens were 98.8% and 79.8%, respectively, while those of urine were 95.1% and 79.2%.

**Figure 4:**
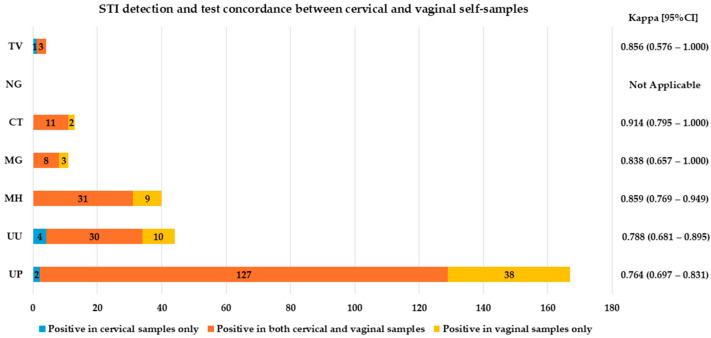
STIs detection and test concordance between cervical and vaginal self-samples. *CT* (*Chlamydia trachomatis)*, *NG* (*Neisseria gonorrhoeae)*, *TV* (*Trichomonas vaginalis)*, *MH* (*Mycoplasma hominis)*, *MG* (*Mycoplasma genitalium)*, *UU* (*Ureaplasma urealyticum)* and *UP* (*Ureaplasma parvum);* CI (confidence interval); Kappa concordance between the self- and clinician-collected cervical samples is presented as follows: slight (0.00 < κ < 0.20), fair (0.21 < κ < 0.40), moderate (0.41 < κ < 0.60), substantial (0.61 < κ < 0.80) and almost perfect (0.81 < κ < 1.00).

**Figure 5:**
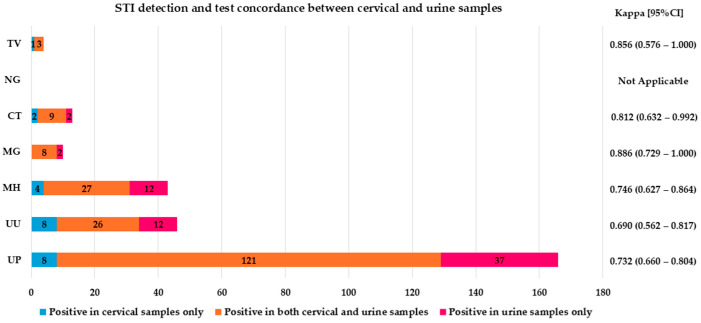
STI detection and test concordance between cervical and urine self-samples. *CT* (*Chlamydia trachomatis)*, *NG* (*Neisseria gonorrhoeae)*, *TV* (*Trichomonas vaginalis)*, *MH* (*Mycoplasma hominis)*, *MG* (*Mycoplasma genitalium)*, *UU* (*Ureaplasma urealyticum)* and *UP* (*Ureaplasma parvum);* CI (confidence interval); Kappa concordance between the self- and clinician-collected cervical samples is presented as follows: slight (0.00 < κ < 0.20), fair (0.21 < κ < 0.40), moderate (0.41 < κ < 0.60), substantial (0.61 < κ < 0.80) and almost perfect (0.81 < κ < 1.00).

The agreement between cervical and self-taken samples for detecting STIs was found to be significant (agreement 88.9% with κ = 0.779 and agreement 86.8% with κ = 0.738 for vaginal and urine specimens, respectively), with almost perfect agreement between urine and vaginal swab specimens (agreement 95% with κ = 0.899) (Figure 4 and Figure 5, and Supplementary Tables S9 and S10).

### 2.6. hrHPV and STI Prevalence in the Different Age Groups

**Figure 6:**
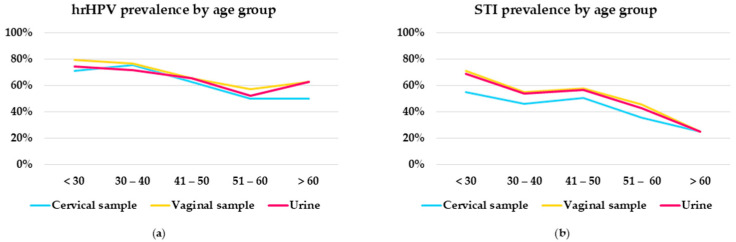
Comparison of hrHPV (**a**) and STI (**b**) prevalence by age group.

Figure 6 shows a comparison between hrHPV and STI prevalence in cervical, vaginal and urine samples across the different age groups of women referred to colposcopy for abnormal cervical cytology. hrHPV prevalence was higher in the younger age groups in all sample types. In cervical samples, the highest hrHPV prevalence (75.5%) was observed in the 30–40 age group of women, whilst hrHPV prevalence was slightly higher in self-collected samples compared to cervical samples among women younger than 30.

Younger women had also a higher prevalence of STIs. In the <30 years of age group, 55.2% of cervical samples, 71.3% of vaginal self-samples and 69.0% of urine were STI-positive. The STIs rates decreased with age, with the lowest prevalence in the >60 years of group, where all sample types showed a prevalence of 25%.

### 2.7. hrHPV and STI Co-Infections

Table 3 shows a comparison between STI prevalence in hrHPV-positive and hrHPV-negative women. For almost all the STIs investigated, the positivity rate among hrHPV-positive women was higher compared to the hrHPV-negative group in all sample types. The difference was statistically significant for *UP* and *MH* in all sample types and for *MG* in vaginal swabs. Details on the statistical analysis conducted are reported in Supplementary Table S11.

The highest prevalence of co-infections with hrHPV and other sexually transmitted pathogens was found in women under 30 years (46.0%), the lowest in women over 60 years (25.0%). Among women with positive and negative colposcopy outcomes, 45.6% and 33.6% of women showed co-infections with hrHPV and STI pathogens, respectively. Rates of co-infections with hrHPV and sexually transmitted pathogens among women with low-grade cervical lesions ranged from 21.4% to 43.8%, while among those with high-grade cervical lesions, the rates ranged from 34.0% to 50.0%. In the group of women with histologically confirmed ≥CIN 2 lesions, 96.6% of women were hrHPV-positive. However, no difference was observed between those with or without concomitant coinfections with other sexually transmitted microorganisms (48.3% vs. 48.3%), as illustrated in Table 4.

## 3. Discussion

In the present study, good analytical agreement was observed between cervical samples and self-collected vaginal and urine samples for the detection of hrHPV, in accordance with data reported by our group in a similar study that investigated the detection of both low- and high-risk HPV genotypes among women referred to colposcopy [12]. The concordance rate of cervical and vaginal specimens was higher than that of cervical and urine samples, consistent with previously reported data [12,28]. Cervical samples confirmed a higher prevalence of single hrHPV infections compared to self-collected samples [12]. These discrepancies may be related to the different anatomical sites of sample collection.

Self-collected specimens have been reported as a valid non-invasive alternative for the detection of hrHPV, contributing to improving women’s participation in HPV screening, and promoted by the World Health Organization (WHO) as a key strategy to accelerate the global fight against cervical cancer [6]. Furthermore, self-collection has shown good acceptability in low-resource settings, improving success in cervical cancer elimination [29,30] by facilitating women’s access to screening, reducing public health costs and alleviating feelings of shame and discomfort by offering privacy to women [29].

The acceptability of self-collection in cervical cancer prevention depends on socio-cultural aspects and women’s perceptions, but it can also be influenced by the attitude of providers who may express doubts about patients’ ability to adequately perform self-collection [31]. Currently, countries such as New Zealand, Australia, the Netherlands, France and Sweden have included self-sampling in their national cervical cancer screening programs [5,11].

The present study has also demonstrated a substantial agreement between self-collected and cervical samples for the detection of STIs in asymptomatic women referred to colposcopy, with the only exception of *UP,* which showed higher positivity rates in self-collected samples. *UP* is commonly found in the genital tract of healthy people, so its pathogenic role can be difficult to prove [32]. Low *MG*, *CT*, *NG* and *TV* positivity rates were detected in this study, probably due to the fact that participants were asymptomatic and recruited when referred to colposcopy for an abnormal Pap smear.

The good analytical sensitivity and specificity for STIs in self-collected samples compared to clinician-collected samples should, however, be further confirmed using diagnostic test accuracy studies to compare the clinical accuracy of different sample types, as recommended for the validation of the hrHPV test by the VALHUDES protocol [33]. The correct assessment of the clinical performance of STI tests, associated with potential false positive and/or false negative results, is essential to understand the cost–benefit of introducing self-sampling as a potential STIs screening method for the general population. Moreover, it is fundamental to understand the clinical relevance of each microorganism and the eventual need for treatment depending on the woman’s clinical presentation. This is particularly true in the case of samples collected from the lower genital tract where more infections from the *Mycoplasmataceae* family have been reported. The improved detection and treatment of STIs may reduce the transmission of these infections, but their over-treatment may be responsible for increased antimicrobial resistance and anxiety in patients receiving a positive result.

In the present study, STIs were more frequently detected among hrHPV-positive than hrHPV-negative women. While persistent hrHPV infections have been demonstrated to be the necessary cause of the onset of cervical precancer and cancer, the potential role of other STIs as cofactors in the development of cervical lesions is still controversial. Some studies have suggested an association between the presence of STIs and a higher risk of developing high-grade cervical lesions [18,19,23,34,35], whilst others have reported no association [24,36].

It is well documented that productive HPV infection is favoured at the ectocervix and that lesion formation begins from the infection of an epithelial stem cell at the transformation zone or endocervix [37]. Changes in vaginal microbiota and infections with other sexually transmitted pathogens have been proposed to act as cofactors in determining HPV-related disease, potentially facilitating viral entry and persistence through chronic cervical inflammation and the ulceration of the upper layers of the cervical epithelium, as well as through a reduction in host cell-mediated immunity [38]. The increased probability of viral entry at the basal layer makes it easier for the virus to persist inside the cells and to advance viral genome integration in the human host cell. Viral integration activates the overproduction of E6 and E7 oncoproteins that are associated with different carcinogenesis pathways [39].

Persistent cervicitis might also enhance the progress of undetected precancerous cervical lesions [25,40]. In fact, previously published reports indicate that cervical carcinogenesis is associated with inflammation [25,40], driven by the hormonal milieu, regulatory cytokines and chemokines, as well as multiple cervicovaginal microorganisms [41]. Inflammation may disrupt the homeostasis of the genital tract, facilitating the entry of virions. The chronic inflammation caused by *Ureaplasma* spp. infections might favour the entry of other microorganisms or induce chromosomal alterations that might lead to the carcinogenesis of epithelial cells [42]. In an earlier study, Lukic et al. postulated that *UU* is related to HPV persistence and early cervical cytological changes [43] through several inflammatory responses, involving the production of reactive oxidative metabolites, the increased expression of cytokines, chemokines and angiogenic factors, decreased cell-mediated immunity and the generation of free radicals [26,35]. Moreover, it is well known that the immunological status of a patient may influence the persistence of HPV infection. The cross-sectional design of the study cannot assess the potential role of persistent STIs and hrHPV infections in cervical lesion progression.

In this study, statistically significant differences were found in the distribution of *UP* and *MH* between hrHPV-positive and hrHPV-negative women in all sample types and of *MG* in vaginal self-samples. Similar results have been previously reported in cervical samples with a significantly higher *UP* infection rate in the HPV-positive group of women compared to the HPV-negative group [27]. In a previous study, Parthenis et al. recruited 345 asymptomatic women participating in routine cervical cancer screening and reported *Ureaplasma* spp. as the most frequently isolated microorganism detected in 30.2% of hrHPV-positive women [44]. Verteramo et al. also showed an increased infection rate of *UU* in HPV-positive women [38]. *Mycoplasma* infections have been linked to “in vitro” chromosomal changes and cell transformation [45,46]. The association between *CT* and cervical cancer has also been widely investigated [23,26,38,47,48]. *CT* might increase susceptibility to HPV causing micro-abrasions or cervical epithelial cells and molecular alterations, facilitating the entry of virions [23,38]. However, in this study, no significant difference was found between *CT, TV* and *UU* distribution in hrHPV-positive women and hrHPV-negative women, probably because of the low frequency of these infections in our population.

The results of the present study further confirm the high prevalence of STIs in young women, as previously described in the literature [49,50]. The risk of acquiring sexually transmitted infections decreases with the decrease in number of sexual encounters, usually associated with an older age. Since this study was conducted in a colposcopy setting, as expected, the prevalence of hrHPV infections was very high as well as the number of women with confirmed cervical lesions. As a result, the high hrHPV positivity rate found in older women may not reflect the trend of hrHPV infections distribution in the general population. The choice of enrolling women in a colposcopy setting was determined by the possibility to investigate the performance of the molecular assays on cervical and self-collected samples on a smaller study population with expected higher positivity rates for hrHPV and STIs; however this may also be considered as a limitation of the present work as it does not provide data on the prevalence of hrHPV and STIs in the general population. Moreover, the limited number of participants and the cross-sectional nature of the study do not allow to completely understand the potential role of hrHPV-STI co-infections in affecting the progression of cervical lesions. Future longitudinal studies, including a larger number of women, will allow to better investigate the possible role of coinfections in cervical lesion development.

In conclusion, the present study confirms that self-taken specimens may be a good alternative for the diagnosis and screening of both hrHPV and other STIs. Due to the increase in the STI rate recently reported, associated with asymptomatic and complicated infections [15], it is important to consider the possibility to include molecular screening for STIs on self-samples collected from women who participate in cervical cancer screening programs. At the same time, improving sexual education, especially among young people, may help to control the transmission of these pathogens and move towards the elimination of STIs and cervical cancer.

## 4. Materials and Methods

### 4.1. Study Design and Sample Collection

For this cross-sectional study, 345 consecutive women from 18 to 69 years (median age 39 years, IQR: 29–46 years) were enrolled at the Colposcopy Clinics of Fondazione IRCCS San Gerardo dei Tintori (Monza, Italy) from May 2017 to September 2024, following the signing of informed consent. Women were referred to colposcopy because of a recent abnormal cervical cytology result. All women were asymptomatic. The following exclusion criteria were applied: immunocompromising or the presence of HIV infection, the state of pregnancy, presumed or ascertained, the presence of immune and autoimmune system diseases, a diagnosis of malignant tumour pathology, and chemotherapy in progress or completed in the 6 months prior to the study.

Prior to the gynaecological examination, all women were asked to autonomously collect a first-void urine sample using a Colli-Pee^®^ device (Novosanis, Belgium) and a vaginal swab using FLOQSwab^®^ 552.80 (Copan Italia Spa, Brescia, Italy).

Before performing the colposcopy, the physician collected a cervical specimen from each woman using an L-shaped Endo/Esocervical FLOQSwab^®^ (Copan Italia Spa, Brescia, Italy) that was immediately resuspended into 20 mL of ThinPrep^®^ PreservCyt^®^ Solution (HOLOGIC™, Marlborough, MA, USA).

Based on the colposcopy findings and clinical judgment, women underwent biopsy and/or treatment with conisation.

The classification of cytological lesions was conducted according to the Bethesda system [51], whilst the histological outcomes were classified according to the WHO histological classification of tumours [52]. Histological lesions worse than cervical intraepithelial neoplasia grade 2 (≥CIN2) were considered high-grade lesions.

This study was conducted following the approval of the Ethics Committee of the University of Milano-Bicocca (Protocol n. 0037320/2017 and update n. 0086409/2018).

### 4.2. Pre-Analytical Sample Processing and Nucleic Acid Extraction

All samples were processed at the Laboratory of Clinical Microbiology of the University of Milano-Bicocca, Monza, Italy. On their arrival at the laboratory, cervical samples were vortexed for 30 s and aliquots of 1.5 mL were dispensed into sterile cryotubes and stored at −20 °C until testing.

First-void urine collected using Colli-Pee^®^ was also shaken on the vortex for 30 s and aliquots of 1.5 mL were stored at −20 °C in sterile cryotubes until testing.

Vaginal self-samples were transported dry at the laboratory where they were suspended in 5.5 mL of ThinPrep^®^ PreservCyt^®^ Solution. Moreover, 1 mL was then dispensed into sterile cryotubes and stored at −20 °C until testing.

In total, 200 μL of all sample types were used to perform nucleic acid extraction using the STARMag 96 × 4 Universal Cartridge Kit (Seegene, Seoul, Republic of Korea) on the MicroLab Nimbus workstation (Hamilton, Reno, NV, USA) with a final elution volume of 100 μL.

### 4.3. HPV and STIs Detection

The Nimbus platform allows the real-time PCR plate preparation of Anyplex™ II HR HPV (Seegene, Seoul, Republic of Korea) and Anyplex™ II STI-7e (Seegene, Seoul, Republic of Korea) assays.

Anyplex™ II HR HPV is a full genotyping HR-HPV assay that individually detects 14 different genotypes of hrHPV (16, 18, 31, 33, 35, 39, 45, 51, 52, 56, 58, 59, 66 and 68) and a cellular gene target by melting curve analysis. The analysis is performed on the CFX96 (Bio-Rad, Hercules, CA, USA) with 5 μL of template DNA in a total volume of 20 μL, as indicated in the manufacturer’s instructions.

Anyplex™ II STI-7e allows the detection of 7 sexually transmitted pathogens, *Chlamydia trachomatis*, *Neisseria gonorrhoeae*, *Trichomonas vaginalis*, *Mycoplasma hominis*, *Mycoplasma genitalium*, *Ureaplasma urealyticum* and *Ureaplasma parvum*. According to the manufacturer’s instructions, the real-time PCR analysis is performed on the CFX96 using 5 μL of template DNA in a total volume of 20 μL.

Data interpretation of the results obtained with both assays was conducted using the Seegene Viewer software (V3 version) according to the manufacturer’s instructions.

Samples that were invalid according to the software interpretation were retested. After two invalid results, samples were excluded from the analysis.

### 4.4. Statistical Analysis

Qualitative and quantitative variables were summarized using absolute and relative (percentage) frequencies and medians (interquartile ranges, IQR), respectively. Concordance between the results of cervical and self-collected specimens with the two assays was evaluated using Cohen’s kappa (κ) statistics and defined as follows: slight (0.00 < κ < 0.20), fair (0.21 < κ < 0.40), moderate (0.41 < κ < 0.60), substantial (0.61 < κ < 0.80) and almost perfect (0.81 < κ < 1.00). Statistical analyses were performed with R software (R Core Team 2021 v. 4.4.0). Statistical significance between positivity rates was calculated using Pearson’s Chi-squared (X^2^) test with Yates’ correction or Fisher’s exact test, as appropriate (Supplementary Tables S2, S5, S6, S8 and S11).

## Figures and Tables

**Table 1:** Clinical data of the study group.

Cytology (*n* = 345)	N	%
HSIL	47	13.6%
ASCH	26	7.5%
LSIL	164	47.5%
ASCUS	86	24.9%
AGC	14	4.1%
NILM	8	2.3%
**Colposcopy (*n* = 345)**		
Positive	127	36.8%
Negative	218	63.2%
**Histological Outcome (*n* = 84)**		
Negative	11	13.1%
CIN 1	13	15.5%
CIN 2	12	14.3%
CIN 3	44	52.4%
Cervical cancer	4	4.8%

HSIL (high-grade squamous intraepithelial lesion); ASCH (atypical squamous cells—cannot exclude HSIL); LSIL (low-grade squamous intraepithelial lesion); ASCUS (atypical squamous cells of undetermined significance); AGC (atypical glandular cell); NILM (negative for intraepithelial lesion or malignancy); CIN 1 (cervical intraepithelial neoplasia grade 1); CIN 2 (cervical intraepithelial neoplasia grade 2); CIN 3 (cervical intraepithelial neoplasia grade 3).

**Table 2:** Distribution of STIs in the three sample types.

	*UP n* (%)	*UU n* (%)	*MH n* (%)	*MG n* (%)	*CT n* (%)	*NG n* (%)	*TV n* (%)
Cervical sample (*n* = 342)	129 (37.7%)	34 (9.9%)	31 (9.1%)	8 (2.3%)	11 (3.2%)	0 (0%)	4 (1.2%)
Vaginal swab (*n* = 342)	165 (48.2%)	40 (11.7%)	40 (11.7%)	11 (3.2%)	13 (3.8%)	0 (0%)	3 (0.9%)
Urine (*n* = 342)	158 (46.2%)	38 (11.1%)	39 (11.4%)	10 (2.9%)	11 (3.2%)	0 (0%)	3 (0.9%)

*UP* (*Ureaplasma parvum*), *UU* (*Ureaplasma urealyticum*), *MH* (*Mycoplasma hominis*), *MG* (*Mycoplasma genitalium*), *CT* (*Chlamydia trachomatis*), *NG* (*Neisseria gonorrhoeae*), *TV* (*Trichomonas vaginalis*).

**Table 3:** Distribution of STIs in hrHPV-positive and -negative women in the three sample types.

		*UP*		*UU*		*MH*		*MG*		*CT*		*NG*		*TV*	
		*n* (%)	*p*	*n* (%)	*p*	*n* (%)	*p*	*n* (%)	*p*	*n* (%)	*p*	*n* (%)	*p*	*n* (%)	*p*
Cervical sample	hrHPV-positive (*n* = 229)	102 (44.5%)	0.0003	27 (11.8%)	0.15	29 (12.7%)	0.0005	8 (3.5%)	0.06	10 (4.4%)	0.11	0 (0%)	1	3 (1.3%)	1
	hrHPV-negative (*n* = 113)	27 (23.9%)		7 (6.2%)		2 (1.8%)		0 (0%)		1 (0.9%)		0 (0%)		1 (0.9%)	
Vaginal swab	hrHPV-positive (*n* = 244)	137 (56.1%)	0.000007	32 (13.1%)	0.27	37 (15.2%)	0.001	11 (4.5%)	0.04	12 (4.9%)	0.12	0 (0%)	1	2 (0.8%)	1
	hrHPV-negative (*n* = 98)	28 (28.6%)		8 (8.2%)		3 (3.1%)		0 (0%)		1 (1.0%)		0 (0%)		1 (1.0%)	
Urine	hrHPV-positive (*n* = 233)	125 (53.6%)	0.00009	30 (12.9%)	0.18	34 (14.6%)	0.006	9 (3.9%)	0.18	10 (4.3%)	0.18	0 (0%)	1	2 (0.9%)	1
	hrHPV-negative (*n* = 109)	33 (30.3%)		8 (7.3%)		5 (4.6%)		1 (0.9%)		1 (0.9%)		0 (0%)		1 (0.9%)	

hrHPV (high-risk Human Papillomavirus), *UP* (*Ureaplasma parvum*), *UU* (*Ureaplasma urealyticum*), *MH* (*Mycoplasma hominis*), *MG* (*Mycoplasma genitalium*), *CT* (*Chlamydia trachomatis*), *NG* (*Neisseria gonorrhoeae*), *TV* (*Trichomonas vaginalis*).

**Table 4:** Distribution of hrHPV and STI co-infection in cervical samples according to clinical data, including age, cytology, colposcopy and histology results of the study population.

	hrHPV+/STI+	hrHPV+/STI−	hrHPV−/STI+	hrHPV−/STI−	Total
**Total Population**	130 (38.0%)	99 (28.9%)	34 (9.9%)	79 (23.1%)	342
**Age in Years (*n* = 342)**					
<30	40 (46.0%)	22 (25.3%)	8 (9.2%)	17 (19.5%)	87
30–40	40 (37.7%)	40 (37.7%)	9 (8.5%)	17 (16.1%)	106
41–50	34 (34.3%)	27 (27.3%)	16 (16.2%)	22 (22.2%)	99
51–60	13 (31.0%)	8 (19.0%)	2 (4.8%)	19 (45.2%)	42
>60	2 (25.0%)	2 (25.0%)	0 (0%)	4 (50.0%)	8
**Cytology (*n* = 342)**					
NILM	0 (0%)	1 (12.5%)	1 (12.5%)	6 (75.0%)	8
ASCUS	26 (30.6%)	26 (30.6%)	10 (11.8%)	23 (27.0%)	85
AGC	3 (21.4%)	2 (14.3%)	1 (7.1%)	8 (57.1%)	14
LSIL	71 (43.8%)	41 (25.3%)	15 (9.3%)	35 (21.6%)	162
ASCH	13 (50.0%)	8 (30.8%)	0 (0%)	5 (19.2%)	26
HSIL	16 (34.0%)	22 (46.8%)	7 (14.9%)	2 (4.3%)	47
**Colposcopy (*n* = 342)**					
Negative	73 (33.6%)	53 (24.4%)	28 (12.9%)	63 (29.1%)	217
Positive	57 (45.6%)	46 (36.8%)	6 (4.8%)	16 (12.8%)	125
**Histology (*n* = 84)**					
<CIN 2	10 (41.7%)	6 (25.0%)	2 (8.3%)	6 (25.0%)	24
≥CIN 2	29 (48.3%)	29 (48.3%)	2 (3.4%)	0 (0%)	60

hrHPV (high-risk Human Papillomavirus), STI (sexually transmitted infection)*,* HSIL (high-grade squamous intraepithelial lesion); ASCH (atypical squamous cells—cannot exclude HSIL); LSIL (low-grade squamous intraepithelial lesion); ASCUS (atypical squamous cells of undetermined significance); AGC (atypical glandular cell); NILM (negative for intraepithelial lesion or malignancy); <CIN 2 (cervical intraepithelial neoplasia inferior to grade 2); ≥CIN 2 (cervical intraepithelial neoplasia grade 2 or worse).

## Data Availability

Final study datasets generated by the study are stored locally and securely at the University of Milano-Bicocca. Anonymized data will be available by request to the corresponding author on a case-by-case basis pending approval by the University of Milano-Bicocca.

## Supplementary Materials

The following supporting information can be downloaded at: https://www.mdpi.com/article/10.3390/ijms26031296/s1.

## Abbreviations

AGC	Atypical glandular cell
ASCH	Atypical squamous cells—cannot exclude HSIL
ASCUS	Atypical squamous cells of undetermined significance
CI	Confidence interval
CIN 1	Cervical intraepithelial neoplasia grade 1
CIN 2	Cervical intraepithelial neoplasia grade 2
CIN 3	Cervical intraepithelial neoplasia grade 3
*CT*	*Chlamydia trachomatis*
ECDC	European Centre for Disease Prevention and Control
hrHPV	High-risk Human Papillomavirus
HPV	Human Papillomavirus
HSIL	High-grade squamous intraepithelial lesion
IARC	International Agency for Research on Cancer
κ	Cohen’s kappa
IQR	Interquartile ranges
LSIL	Low-grade squamous intraepithelial lesion
*MG*	*Mycoplasma genitalium*
*MH*	*Mycoplasma hominis*
*NG*	*Neisseria gonorrhoeae*
NILM	Negative for intraepithelial lesion or malignancy
STI	Sexually transmitted infection
*TV*	*Trichomonas vaginalis*
*UP*	*Ureaplasma parvum*
*UU*	*Ureaplasma urealyticum*
VALHUDES	Validation of Human Papillomavirus Assays and Collection Devices for Self-samples and Urine Samples
WHO	World Health Organization
