# Supplementary material for: Molecular Detection of Human Papillomavirus (HPV) and Other Sexually Transmitted Pathogens in Cervical and Self-Collected Specimens
Source: Int J Mol Sci. 2025 Feb 3;26(3):1296. doi: 10.3390/ijms26031296 (PMC11818694; doi:10.3390/ijms26031296)
Supplement: Supplementary file 1 [file ijms-26-01296-s001.zip › ijms-3412558-supplementary.pdf]

Supplementary Materials

Supplementary Table S1. Single and multiple infections caused by hrHPV in different samples.

	**Cervical sample n. (%)**<br>**n = 229**	**Vaginal swab n. (%)**<br>**n = 244**	**Urine n. (%)**<br>**n = 233**
Single hrHPV	129 (56.3%)	120 (49.2%)	119 (51.1%)
2 hrHPV	58 (25.3%)	77 (31.6%)	65 (27.9%)
3 hrHPV	30 (13.1%)	24 (9.8%)	27 (11.6%)
More than 3 hrHPV	12 (5.2%)	23 (9.4%)	22 (9.4%)

Supplementary Table S2. Statistical analysis relative to the distribution of single and multiple infections caused by hrHPV in different samples

		**Cervical sample vs Vaginal swab**	**Cervical sample vs Urine**
**Single hrHPV**	**Test**	**X^2 test with Yates correction**	**X^2 test with Yates correction**
	**df**	**1**	**1**
	**X^2**	**2.1451**	**1.0818**
	**p-value**	**0.143**	**0.2983**
	**significativity**	**none**	**none**
**2 hrHPV**	**Test**	**X^2 test with Yates correction**	**X^2 test with Yates correction**
	**df**	**1**	**1**
	**X^2**	**1.9529**	**0.26987**
	**p-value**	**0.1623**	**0.6034**
	**significativity**	**none**	**none**
**3 hrHPV**	**Test**	**X^2 test with Yates correction**	**X^2 test with Yates correction**
	**df**	**1**	**1**
	**X^2**	**0.94288**	**0.12444**
	**p-value**	**0.3315**	**0.7243**
	**significativity**	**none**	**none**
**More than 3 hrHPV**	**Test**	**X^2 test with Yates correction**	**X^2 test with Yates correction**
	**df**	**1**	**1**
	**X^2**	**2.441**	**2.4063**
	**p-value**	**0.1182**	**0.1208**
	**significativity**	**none**	**none**

Supplementary Table S3. Type-specific agreement and test concordance between cervical and vaginal self-samples.

	HPV type	+/+[1]	+/-	-/+	-/-	Agreement [%]	Kappa[2] [95% CI]
	hrHPV	227	2	17	96	94.4	0.870 (0.814 - 0.927)
	HPV16	79	1	7	255	97.7	0.936 (0.893 - 0.980)
	HPV18	18	0	4	320	98.8	0.894 (0.791 - 0.997)
	HPV31	44	2	9	287	96.8	0.870 (0.795 - 0.945)
	HPV33	10	5	3	324	97.7	0.702 (0.506 - 0.898)
	HPV35	7	0	4	331	98.8	0.772 (0.556 - 0.988)
**Total**	HPV39	19	1	2	320	99.1	0.922 (0.835 - 1.000)
**population**	HPV45	10	2	5	325	98.0	0.730 (0.539 - 0.922)
**(n = 342)**	HPV51	26	0	10	306	97.1	0.823 (0.717 - 0.929)
	HPV52	27	3	3	309	98.2	0.890 (0.804 - 0.977)
	HPV56	25	0	4	313	98.8	0.920 (0.842 - 0.998)
	HPV58	24	2	8	308	97.1	0.812 (0.699 - 0.925)
	HPV59	19	0	4	319	98.8	0.899 (0.800 - 0.997)
	HPV66	33	1	8	300	97.4	0.865 (0.779 - 0.951)
	HPV68	27	2	8	305	97.1	0.828 (0.724 - 0.932)

Supplementary Table S4. Type-specific agreement and test concordance between cervical and urine self-samples.

	HPV type	+/+[1]	+/-	-/+	-/-	Agreement [%]	Kappa[2] [95% CI]
	hrHPV	214	15	19	94	90.1	0.773 (0.701 - 0.845)
	HPV16	68	12	11	251	93.3	0.812 (0.738 - 0.886)
	HPV18	14	4	3	321	98.0	0.789 (0.638 - 0.941)
	HPV31	43	2	6	291	97.7	0.901 (0.834 - 0.969)
	HPV33	10	5	3	324	97.7	0.702 (0.506 - 0.898)
	HPV35	7	0	6	329	98.2	0.692 (0.459 - 0.924)
**Total**	HPV39	16	4	4	318	97.7	0.788 (0.645 - 0.930)
**population**	HPV45	9	3	2	328	98.5	0.775 (0.584 - 0.966)
**(n = 342)**	HPV51	23	3	11	305	95.9	0.745 (0.617 - 0.872)
	HPV52	24	6	2	310	97.7	0.775 (0.584 - 0.966)
	HPV56	25	0	5	312	98.5	0.901 (0.816 - 0.987)
	HPV58	21	5	8	308	96.2	0.743 (0.609 - 0.877)
	HPV59	18	1	4	319	98.5	0.870 (0.758 - 0.982)
	HPV66	31	3	11	297	95.9	0.793 (0.689 - 0.897)
	HPV68	23	6	17	296	93.3	0.630 (0.492 - 0.769)

hr (high-risk); HPV (Human Papillomavirus); CI (confidence interval); n (number). [1] +/+ positive on self- and cervical samples, +/- positive only on cervical samples, -/+ positive only on self-samples, -/- negative on both sample types. [2] Kappa concordance between the self- and clinician-collected cervical samples is presented as follows: slight ($0.00 < \kappa < 0.20$), fair ($0.21 < \kappa < 0.40$), moderate ($0.41 < \kappa < 0.60$), substantial ($0.61 < \kappa < 0.80$) and almost perfect ($0.81 < \kappa < 1.00$).

Supplementary Figure S1. Prevalence of STI pathogens in different samples.

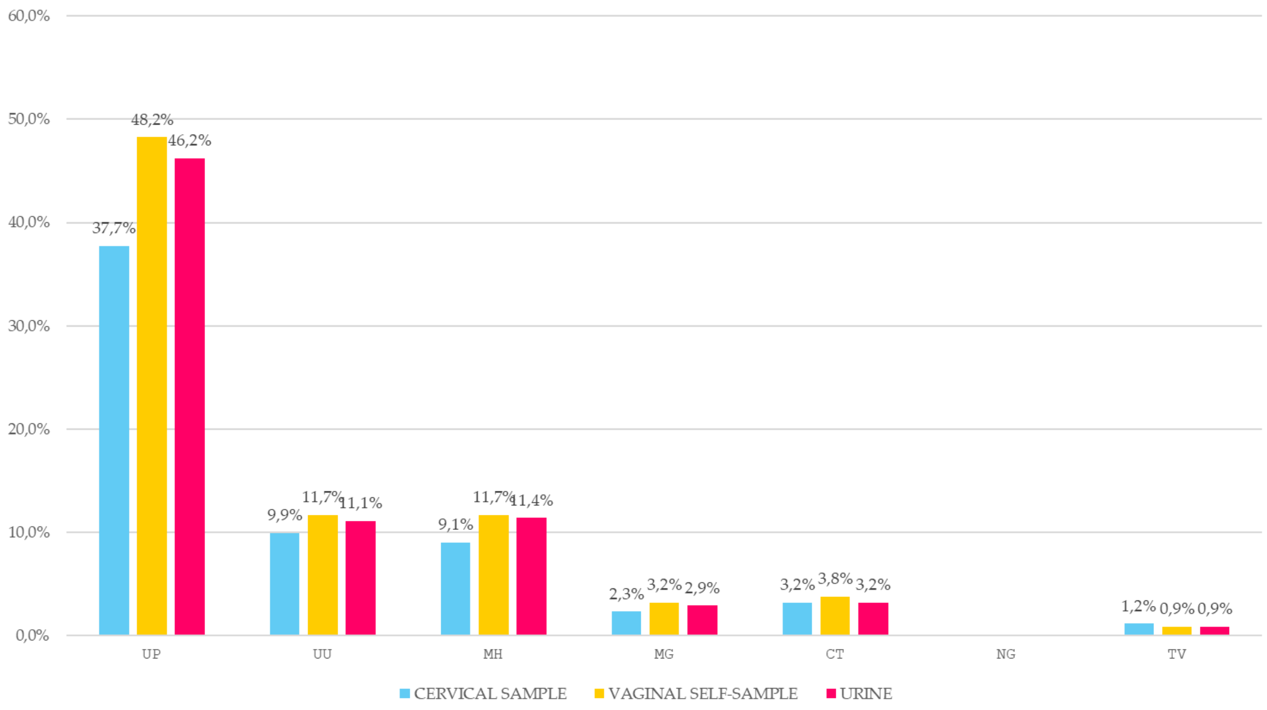

Supplementary Table S5. Tests used to calculate statistical significance differences between positivity rates of UP, UU, MH and MG in the three sample types.

	UP						*UU*						*MH*						*MG*					
	**Cervical sample**			**Vaginal swab**			**Cervical sample**			**Vaginal swab**			**Cervical sample**			**Vaginal swab**			**Cervical sample**			**Vaginal swab**		
	**Test**	**df**	**X^2**	**Test**	**df**	**X^2**	**Test**	**df**	**X^2**	**Test**	**df**	**X^2**	**Test**	**df**	**X^2**	**Test**	**df**	**X^2**	**Test**	**df**	**X^2**	**Test**	**df**	**X^2**
**Cervical sample**	-	-	-	X^2 test with Yates correction	1	7.3077	-	-	-	X^2 test with Yates correction	1	0.3788	-	-	-	X^2 test with Yates correction	1	1.0058	-	-	-	X^2 test with Yates correction	1	0.2165
**Vaginal swab**	X^2 test with Yates correction	1	7.3077	-	-	-	X^2 test with Yates correction	1	0.3788	-	-	-	X^2 test with Yates correction	1	1.0058	-	-	-	X^2 test with Yates correction	1	0.2165	-	-	-
**Urine**	X^2 test with Yates correction	1	4.7065	X^2 test with Yates correction	1	0.2112	X^2 test with Yates correction	1	0.1397	X^2 test with Yates correction	1	0.0145	X^2 test with Yates correction	1	0.7798	X^2 test with Yates correction	1	0	X^2 test with Yates correction	1	0.0571	X^2 test with Yates correction	1	0

df (degrees of freedom); X^2 (Chi-squared); OR (odds ratio); *UP* (*Ureaplasma parvum*); *UU* (*Ureaplasma urealyticum*); *MH* (*Mycoplasma hominis*) and *MG* (*Mycoplasma genitalium*).

Supplementary Table S6. Tests used to calculate statistical significance differences between positivity rates of CT, NG and TV in the three sample types.

	CT						*NG*						*TV*					
	**Cervical sample**			**Vaginal swab**			**Cervical sample**			**Vaginal swab**			**Cervical sample**			**Vaginal swab**		
	**Test**	**df**	**X^2**	**Test**	**df**	**X^2**	**Test**	**df**	**X^2**	**Test**	**df**	**X^2**	**Test**	**CI**	**OR**	**Test**	**CI**	**OR**
**Cervical sample**	-	-	-	X^2 test with Yates correction	1	0.0432	-	-	-	X^2 test with Yates correction	1	0	-	-	-	Fisher's exact test	95%	1.3367
**Vaginal swab**	X^2 test with Yates correction	1	0.0432	-	-	-	X^2 test with Yates correction	1	0	-	-	-	Fisher's exact test	95%	1.3367	-	-	-
**Urine**	X^2 test with Yates correction	1	0	X^2 test with Yates correction	1	0.0432	X^2 test with Yates correction	1	0	X^2 test with Yates correction	1	0	Fisher's exact test	95%	1.3367	Fisher's exact test	95%	1

CT (*Chlamydia trachomatis*); *NG* (*Neisseria gonorrhoeae*) and *TV* (*Trichomonas vaginalis*).

Supplementary Table S7. Single and multiple STI infections in different samples.

	**Cervical sample n. (%) n = 164**	**Vaginal swab n. (%) n = 198**	**Urine n. (%) n = 193**
Single STI	122 (74.4%)	143 (72.2%)	141 (73.1%)
2 STIs	32 (19.5%)	38 (19.2%)	39 (20.2%)
More than 2 STIs	10 (6.1%)	17 (8.6%)	13 (6.7%)

Supplementary Table S8. Statistical analysis relative to the distribution of single and multiple STI infections in different samples.

		**Cervical sample vs Vaginal swab**	**Cervical sample vs Urine**
**Single STI**	**Test**	X^2 test with Yates correction	X^2 test with Yates correction
	**df**	1	1
	**X^2**	0.11863	0.027051
	**p-value**	0.7305	0.8694
	**significativity**	none	none
**2 STI**	**Test**	X^2 test with Yates correction	X^2 test with Yates correction
	**df**	1	1
	**X^2**	0,000000000000000000000000000002791	0.00095662
	**p-value**	1	0.9753
	**significativity**	none	none
**More than 2 STIs**	**Test**	X^2 test with Yates correction	X^2 test with Yates correction
	**df**	1	1
	**X^2**	0.48454	0.00081083
	**p-value**	0.4864	0.9773
	**significativity**	none	none

Supplementary Table S9. Statistical analysis relative to the distribution of single and multiple STI infections in different samples.

	**STI**	+/+[1]	+/-	-/+	-/-	**Agreement [%]**	**Kappa[2] [95% CI]**
**Total population (n = 342)**	STI	162	2	36	142	88.9	0.779 (0.714 - 0.844)
	UP	127	2	38	175	88.3	0.764 (0.697 - 0.831)
	UU	30	4	10	298	95.9	0.788 (0.681 - 0.895)
	MH	31	0	9	302	97.4	0.859 (0.769 - 0.949)
	MG	8	0	3	331	99.1	0.838 (0.657 - 1.000)
	CT	11	0	2	329	99.4	0.914 (0.795 - 1.000)
	NG	0	0	0	342	100	Not Applicable
	TV	3	1	0	338	99.7	0.856 (0.576 - 1.000)

Supplementary Table S10. Type-specific agreement and test concordance between cervical and urine self-samples.

	**STI**	+/+[1]	+/-	-/+	-/-	**Agreement [%]**	**Kappa[2] [95% CI]**
**Total population (n = 342)**	STI	156	8	37	141	86.8	0.738 (0.668 - 0.808)
	UP	121	8	37	176	86.8	0.732 (0.660 - 0.804)
	UU	26	8	12	296	94.1	0.690 (0.562 - 0.817)
	MH	27	4	12	299	95.3	0.746 (0.627 - 0.864)
	MG	8	0	2	332	99.4	0.886 (0.729 - 1.000)
	CT	9	2	2	329	98.8	0.812 (0.632 - 0.992)
	NG	0	0	0	342	100	Not Applicable
	TV	3	1	0	338	99.7	0.856 (0.576 - 1.000)

STI (sexually transmitted infection); *CT* (*Chlamydia trachomatis*), *NG* (*Neisseria gonorrhoeae*), *TV* (*Trichomonas vaginalis*), *MH* (*Mycoplasma hominis*), *MG* (*Mycoplasma genitalium*), *UU* (*Ureaplasma urealyticum*) and *UP* (*Ureaplasma parvum*); CI (confidence interval); n (number). [1] +/+ positive on self- and cervical samples, +/- positive only on cervical samples, -/+ positive only on self-samples, -/- negative on both sample types. [2] Kappa concordance between the self- and clinician-collected cervical samples is presented as follows: slight (0.00 < κ < 0.20), fair (0.21 < κ < 0.40), moderate (0.41 < κ < 0.60), substantial (0.61 < κ < 0.80) and almost perfect (0.81 < κ < 1.00).

Supplementary Table S11. Tests used to calculate statistical significance differences between positivity rates of STI pathogens in hrHPV-positive and negative women in the three sample types.

	UP			*UU*			*MH*			*MG*			*CT*			*NG*			*TV*		
	**test**	**df**	**X^2**	**test**	**df**	**X^2**	**test**	**CI**	**OR**	**test**	**CI**	**OR**	**test**	**CI**	**OR**	**test**	**CI**	**OR**	**test**	**CI**	**OR**
**Cervical samples (n = 342)**	X^2 test with Yates correction	1	12.8665	X^2 test with Yates correction	1	2.0581	Fisher's exact test	95%	8.0168	Fisher's exact test	95%	Inf	Fisher's exact test	95%	5.0981	Fisher's exact test	95%	0	Fisher's exact test	95%	1.4851
**Vaginal swab (n = 342)**	X^2 test with Yates correction	1	20.2036	X^2 test with Yates correction	1	1.215	Fisher's exact test	95%	5.6396	Fisher's exact test	95%	Inf	Fisher's exact test	95%	5.19	Fisher's exact test	95%	0	Fisher's exact test	95%	0.8022
**Urine (n = 342)**	X^2 test with Yates correction	1	15.3946	X^2 test with Yates correction	1	1.778	Fisher's exact test	95%	3.5432	Fisher's exact test	95%	4.3256	Fisher's exact test	95%	4.8283	Fisher's exact test	95%	0	Fisher's exact test	95%	0.9353

Supplementary Table S12. Distribution of hrHPV-positive cervical samples according to clinical data, including age, cytology, colposcopy and histology results of the study population.

	**hrHPV positivity on cervical sample n/N (%)**
**Age in years (n = 342)**	
<30	62/87 (71.3%)
30-40	80/106 (75.5%)
41-50	61/99 (61.6%)
51-60	21/42 (50.0%)
>60	4/8 (50.0%)
**Cytology (n = 342)**	
NILM	1/8 (12.5%)
ASCUS	52/85 (61.2%)
AGC	5/14 (35.7%)
LSIL	112/162 (69.1%)
ASCH	21/26 (80.8%)
HSIL	38/47 (80.9%)
**Colposcopy (n = 342)**	
Negative	126/217 (58.0%)
Positive	103/125 (82.4%)
**Histology (n = 84)**	
< CIN 2	16/24 (66.7%)
≥ CIN 2	58/60 (96.7%)

n/N (number of hrHPV+/total cohort
